# Is Depression or Apathy Playing a Key Role in Predicting Financial Capacity in Parkinson’s Disease with Dementia and Frontotemporal Dementia?

**DOI:** 10.3390/brainsci11060785

**Published:** 2021-06-14

**Authors:** Vaitsa Giannouli, Magda Tsolaki

**Affiliations:** 1 Department of Neurology, School of Medicine, Faculty of Health Sciences, Aristotle University of Thessaloniki, 54634 Thessaloniki, Greece; tsolakim1@gmail.com; 2 School of Psychology, University of Western Macedonia, 53100 Florina, Greece

**Keywords:** financial capacity, Parkinson disease, frontotemporal dementia, apathy, depression

## Abstract

(1) Background: Depression and apathy both affect cognitive abilities, such as thinking, concentration and making decisions in young and old individuals. Although apathy is claimed to be a “core” feature of Parkinson’s disease (PD) and frontotemporal dementia (FTD), it may occur in the absence of depression and vice versa. Thus, the aim of this study is to explore whether depression or apathy better predict financial capacity performance in PD and FTD as well as in nondemented participants. (2) Methods: Eighty-eight participants divided into three groups (PD, FTD and non-demented participants) were examined with the Mini-Mental State Examination (MMSE) and the Legal Capacity for Property Law Transactions Assessment Scale (LCPLTAS)—Full and short form. The Geriatric Depression Scale informant version (GDS-15) and the Irritability-Apathy Scale (IAS) we completed by caregivers. (3) Results: The results indicated that both PD and FTD patients’ general cognitive functioning and financial capacity performance is negatively influenced by apathy and not by depression. (4) Conclusions: Differences in financial capacity performance indicate that apathy should not be disregarded in clinical assessments. Further studies on larger PD and FTD populations are necessary in order to investigate the decisive role of mood factors on financial capacity impairment.

## 1. Introduction

Apathy in Parkinson’s disease (PD) is a frequently reported neuropsychiatric symptom associated with lower mean Mini-Mental State Evaluation (MMSE) scores and an increased risk of co-morbid depression [1]. The same applies for frontotemporal dementia (FTD), as it is found to be a common symptom in this group of patients [2]. Financial capacity has been found to be negatively influenced by depressive symptoms are present in PD [3], in vascular dementia [4], in mild Alzheimer’s disease [5], and in amnestic mild cognitive impairment [6].

Therefore, the role of depression is thoroughly investigated, leaving unclear whether apathy either seen as is a distinct syndrome or merely a symptom of depression may influence cognition, and more specifically, a little investigated complex cognitive capacity; that of financial capacity. Financial capacity consists of a variety of activities and specific skills, such as performance skills relating to arithmetic counting coins-currency, paying bills, and judgment/decision making skills, but relevant research attempts do not encompass PD and FTD.

Based on the common finding that depressed patients often have symptoms of apathy, the aim of this study is not only to compare financial capacity performance in PD and FTD patients with similar demographics, but to investigate whether depression or apathy predict financial capacity impairments.

## 2. Materials and Methods

The current study assessed eighty-eight older adults (45 women, 43 men) from Greece. Overall, 30 participants were patients with PD, under levodopa treatment for at least two years, who met the criteria for dementia (PDD), based on DSM-IV criteria for dementia and clinical recommendations for PDD diagnosis, such as a diagnosis of PD which developed prior to the onset of dementia, decreased global cognitive efficiency, cognitive deficiency that is severe enough to impair daily life, and the impairment of one or more cognitive domains (attention, cognitive function, visuo-constructive ability, and/or memory [7]. Additionally, 28 patients with frontotemporal disease (FTD) and more specifically possible behavioral variant (bvFTD) (demonstrating three of the following features-criteria; (1) behavioral disinhibition, (2) apathy or inertia, (3) loss of sympathy or empathy, (4) perseverative, stereotyped or compulsive/ritualistic, (5) hyperorality and dietary changes, and (6) executive dysfunction with relative sparing of memory and visuospatial functions) were examined. The 15-item informant version of the Geriatric Depression Scale (GDS-15) was administered to caregivers and family members for an objective way of gathering information about depressive symptomatology [8]. In addition to that, the same knowledgeable informant for each person with dementia completed the Irritability-Apathy Scale (IAS), which measures apathy with the IAS-apathy subscale, comprised of 5 items, rated each on a 5-point Likert scale (ranging from 1 = much more interest to 5 = much less interest), with higher scores on the IAS-apathy subscale, indicating more severe apathy [9].

Thirty participants matched with the two patient groups in age, sex, and level of education, coming from a larger pool of data, were also tested as a control group. PDD patients, FTD patients and healthy controls were excluded from the study if comorbid conditions occurred, such as concomitant serious medical illness (including significant visual or auditory impairment not corrected sufficiently by visual/auditory aids), and a history of prior or current substance abuse and neurosurgical interventions. The exclusion criterion for the group of healthy controls and patients was a history of major psychiatric condition (e.g., schizophrenia), as such conditions could interfere with the testing procedure. All participant groups as young adults had been autonomous/responsible for administrative and financial management, so a previous experience with financial affairs did occur for the whole sample and participants were not unfamiliar with financial activities according to their self-reports and their caregivers’ responses during the history taking.

Global cognition was directly assessed with the Mini-Mental State Examination (MMSE), a test that was selected to be included in the analyses due to its importance in the Greek neuropsychological assessment protocols. Financial capacity was assessed with the Greek version of the Legal Capacity for Property Law Transactions Assessment Scale (LCPLTAS), full and short form (please also see Reference [10], for the full neuropsychological evaluation with a battery of standardized tests for the two groups of patients). LCPLTAS is the only standardized tool in use in Greece based on the conceptual model for financial capacity of Marson’s et al. [11]. The LCPLTAS (full and short form) consists of 7 domains: (1) basic monetary skills, (2) cash transactions, (3) bank statement management, (4) bill payment, (5) financial conceptual knowledge, (6) financial decision making, and (7) knowledge of personal assets.

A “caregiver” who interacted systematically for at least one year with the older adult on a regular-everyday basis was also examined during a separate session. The “caregivers” could be a spouse or an adult–child (controls: *n* = 19 spouses, *n* = 11 adult–children; FTD patients: *n* = 21 spouses, *n* = 7 adult–children; PD patients: *n* = 18 spouses, *n* = 12 adult–children). The «caregivers» (partners, children) of the healthy group had matched demographics with the caregivers of FTD and PD patients regarding age [F(2, 87) = 2.486, *p* = 0.089], socioeconomic status (all participants were of middle level as measured by yearly income), and years of education [F(2, 87) = 1.008, *p* = 0.369]. At this point, we have to mention that all groups of participants had similar sociodemographic characteristics (were all in a relationship-marriage, with adult children, and had an average yearly household income), as indicated by themselves during the completion of the demographics questionnaire.

Ethical approval was obtained from the Research Ethics Committee of the School of Medicine, Aristotle University of Thessaloniki, Thessaloniki, Greece (2/27.3.2013).

Statistical analyses were performed using IBM SPSS Statistics version 21. The statistics mean (M), standard deviation (SD) for all the abovementioned variables were computed, one-way analysis of variance (ANOVA) was performed regarding the FTD, PDD and healthy groups in search of possible differences in MMSE, IAS-apathy scores, LCPTLAS full form and short form and Tamhane’s T2 was applied as a post hoc test to see which diagnostic groups differed from each other in terms of MMSE, LCPTLAS full form and short form and IAS-apathy scores. “Enter” regression analysis was conducted with LCPTLAS score as dependent variable and IAS-apathy scores and GDS—15 as the independent predictors.

## 3. Results

We compared PD patients, FTD patients and the control group using one-way ANOVA on neuropsychological performance and “Enter” method regression analyses of depression and apathy indices on financial capacity and MMSE performance.

When ANOVA was applied, statistically significant differences and large effect sizes were found for the MMSE scores [F(2, 87) = 66.993, *p* < 0.001, η^2^ = 0.611], but also the financial capacity as examined by LCPTLAS full form [F(2, 87) = 53.704, *p* < 0.001, η^2^ = 0.558], the LCPTLAS short form [F(2, 87) = 52.947, *p* < 0.001, η^2^ = 0.396], and the IAS-apathy scores [F(2, 87) = 27.896, *p* < 0.001, η^2^ = 0.833]. Post hoc Tamhane’s T2 (applied due to unequal analyses) indicated that the three groups differed from each other, as they had statistically different scores in MMSE, IAS-apathy scores, and in LCPTLAS short form as well as full form (see Table 1). Post hoc analyses revealed that the MMSE (*p* < 0.001 and *p* < 0.001), LCPTLAS full form (*p* < 0.001 and *p* < 0.001), LCPTLAS short form (*p* < 0.001 and *p* < 0.001), and IAS-apathy scores (*p* < 0.001 and *p* < 0.001) were different between healthy individuals and PDD patients and FTD patients, respectively. Additionally, post hoc analyses revealed that the MMSE (*p* < 0.001 and *p* < 0.001), LCPTLAS full form (*p* < 0.001 and *p* < 0.001), LCPTLAS short form (*p* < 0.001 and *p* < 0.001), and IAS-apathy scores (*p* < 0.001 and *p* < 0.001) were different between PDD patients and healthy participants and FTD patients, respectively.

Linear regression model, “Enter” method indicated that only apathy predicted financial capacity scores (R = 0.821; R^2^ = 0.673; see Table 2) and a similar finding was found when MMSE was the dependent variable (R = 0.815; R^2^ = 0.664; see Table 1 where B stands for the unstandardized regression coefficient and beta is the standardized one).

## 4. Discussion

Patients performed more poorly compared to their healthy peers (2–2.5 SDs lower from the healthy controls’ mean performance) on financial capacity, as measured by the LCPTLAS scores. Although both depression and apathy are expected to have a negative influence on global cognition as measured with MMSE, these preliminary findings provide support for the importance of apathy as the best predictor of financial capacity in PDD and FTD. Moreover, we found that apathy seems to play the most important role, but so far, no research has focused on this, therefore rendering necessary in assessments in legal as well as medical settings also to include this disregarded variable. Given that nowadays apathy is regarded as a transdiagnostic syndrome influencing goal-directed activity, this study supports previous findings examining separately the relationship of underlying brain areas linked to apathy and the brain substrates of financial capacity which seem to coincide, such as medial frontal cortex and subcortical structures among others, both in PDD and FTD [12,13]. This is of interest in forensic neuropsychology, as it may assist us in making relevant financial capacity predictions [14].

A direct measurement of apathy and depression was avoided, and informant reports were preferred, as apathy usually is accompanied by diminished self-awareness, so changes typically are noticed and brought to the attention of clinicians by caregivers [15].

One of the major limitations of this study is that, given the fact that this is a new topic under investigation, we did not take into consideration possible unknown covariates (e.g., motivation and/or malingering during the neuropsychological assessment), while issues of ecological validity may arise, given that the study design relied on third-raters evaluations coming from the “caregivers”. Although caregivers’ reports are considered a legitimate form of inquiry when third-party evaluations are made, there is always the risk of overestimation and/or underestimation [16,17], thus additional objective measures, other than caregivers’ views, could be incorporated (e.g., expert observers-clinicians monitoring the patients in an everyday basis experimental design). Future research should further explore if this apathy-depression debate in PDD and FTD patients and the detrimental effects of apathy on financial capacity can be tracked on exactly the same brain dysfunctions in the two disorders. Moreover, the utility of other neuropsychological tests, other than MMSE, should be further evaluated when comparing PDD and FTD patients with other groups of older patients, such as Alzheimer’s disease, dementia with Lewy bodies and other neurocognitive disorders [18,19].

## Figures and Tables

**Table 1:** Demographics and scores on MMSE, LCPTLAS full and short form, IAS, and GDS-15, of PDD patients, FTD patients and healthy controls.

		Mean	SD	Minimum and Maximum Values
Age	Healthy	76.00	8.70	58–91
	PD	76.03	8.85	57–91
	FTD	75.78	8.41	57–88
Education in years	Healthy	8.50	3.75	4–16
	PD	8.60	4.20	3–18
	FTD	6.67	2.91	1–16
MMSE	Healthy	29.46	.62	28–30
	PD	22.50	3.57	14–26
	FTD	18.39	5.36	10–28
GDS-15	Healthy	8.64	0.99	7–10
	PD	7.95	4.40	0–15
	FTD	6.16	3.55	1–14
IAS-apathy subscale	Healthy	3.86	0.57	3–5
	PD	10.18	6.86	1–24
	FTD	15.21	7.41	2–25
LCPTLAS short form	Healthy	142.73	1.48	138–144
	PD	94.53	38.51	28–144
	FTD	54.82	41.85	0–130
LCPTLAS full form	Healthy	209.86	2.04	204–212
	PD	141.83	54.09	53–212
	FTD	82.39	61.43	0–191

**Table 2:** Results from regression analyses for apathy and depression.

Model 1	*B*	Beta	*t*	*P*
(Constant)	135.008		13.915	<0.001
ΙAS (Apathy subscale)	−5.423	−0.827	−10.641	<0.001
GDS-15	1.725	0.126	1.621	0.111
Dependent Variable: LCPTLAS (financial capacity)				
**Model 1**	***B***	**Beta**	***t***	***P***
(Constant)	27.358		23.420	<0.001
ΙAS (Apathy subscale)	−0.639	−0.820	−10.412	<0.001
GDS-15	0.246	0.151	1.918	0.060

Note: Dependent Variable: MMSE.

## Data Availability

The data presented in this study are available on request from the corresponding author.
